# *NSD3-NUTM1*-rearranged carcinoma of the median neck/thyroid bed developing after recent thyroidectomy for sclerosing mucoepidermoid carcinoma with eosinophilia: report of an extraordinary case

**DOI:** 10.1007/s00428-021-03103-8

**Published:** 2021-04-23

**Authors:** Abbas Agaimy, Lars Tögel, Robert Stoehr, Norbert Meidenbauer, Sabine Semrau, Arndt Hartmann, Konstantinos Mantsopoulos

**Affiliations:** 1 Institute of Pathology, Friedrich-Alexander University Erlangen-Nürnberg (FAU), Krankenhausstrasse 8-10, 91054 Erlangen, Germany; 2 Departments of Internal Medicine 5 (Medical Oncology and Hematology), Friedrich-Alexander University Erlangen-Nürnberg (FAU), Erlangen, Germany; 3 Department of Radiation Oncology, Friedrich-Alexander University Erlangen-Nürnberg (FAU), Erlangen, Germany; 4 Department of Otorhinolaryngology, Head and Neck Surgery, Friedrich-Alexander-University Erlangen-Nürnberg (FAU), Erlangen, Germany

**Keywords:** NUT carcinoma, NUTM1, NSD3, Midline carcinoma, Sclerosing mucoepidermoid carcinoma with eosinophilia, Thyroid gland, Head and neck

## Abstract

Sclerosing mucoepidermoid carcinoma with eosinophilia (SMECE) is an exceedingly rare low-grade thyroid malignancy of unknown histogenesis. NUT carcinoma is another rare, highly aggressive neoplasm with predilection for the midline, defined by recurrent *NUTM1* fusions. The bromodomain family genes (*BRD4* or *BRD3*) and rarely *NSD3*, *ZNF532*, or others are known fusion partners. We describe an extraordinary case of a 42-year-old female with a thyroid SMECE treated by thyroidectomy and neck dissection. She presented 6 months later with extensive midline recurrence encasing/compressing the trachea. Biopsy revealed poorly differentiated carcinoma with abrupt squamous differentiation, suggestive of NUT carcinoma. Immunohistochemistry confirmed expression of monoclonal NUT antibody. Targeted RNA sequencing revealed the *NSD3-NUTM1* fusion in the NUT carcinoma, but not in the SMECE. This unique case highlights unusual sequential origin of two exceptionally rare entities at same anatomic site and underlines the necessity of sampling unexpectedly aggressive recurrences of otherwise indolent malignancies.

## Introduction

Sclerosing mucoepidermoid carcinoma with eosinophilia (SMECE) is an exceptionally rare low-grade variant of thyroid carcinomas. Since its first description in 1991, < 60 cases have been reported [1–3]. The disease presents more frequently in females, on average in the 5th decade. SMECE frequently is associated with chronic lymphocytic background Hashimoto-type thyroiditis [2, 3]. Although some similarities to salivary gland mucoepidermoid carcinoma are observed, the current concept is that SMECE of the thyroid is distinct from salivary-type mucoepidermoid carcinoma [2, 3]. SMECE is generally considered an indolent neoplasm, but one-third of cases may recur locally and/or metastasize [2, 3]. The molecular pathogenesis of SMECE remains unknown [3].

NUT carcinoma (synonym: NUT midline carcinoma) is another rare highly aggressive epithelial malignancy with predilection for midline structures (upper aerodigestive tract, thymus, and mediastinum), predominantly affecting children, adolescents, and young adults but may occur at any age. To date, < 200 cases have been reported [4, 5]. NUT carcinoma is characterized by gene rearrangements involving the nuclear protein in testis (*NUT*; also known as *NUTM1*) gene at 15q14 which is most commonly fused to one of the bromodomain family members, mainly *BRD4* at 19.p13 and less frequently *BRD3* or *NSD3* [4, 5]. Availability of small molecules targeting the bromodomain pathways underlines the importance of correctly recognizing NUT carcinoma [6, 7]. We herein describe clinicopathological and molecular characteristics of an unusual *NSD3-NUTM1*-rearranged NUT carcinoma developing in the thyroid bed 6 months after thyroidectomy for SMECE.

## Clinical history

**Fig. 1:**
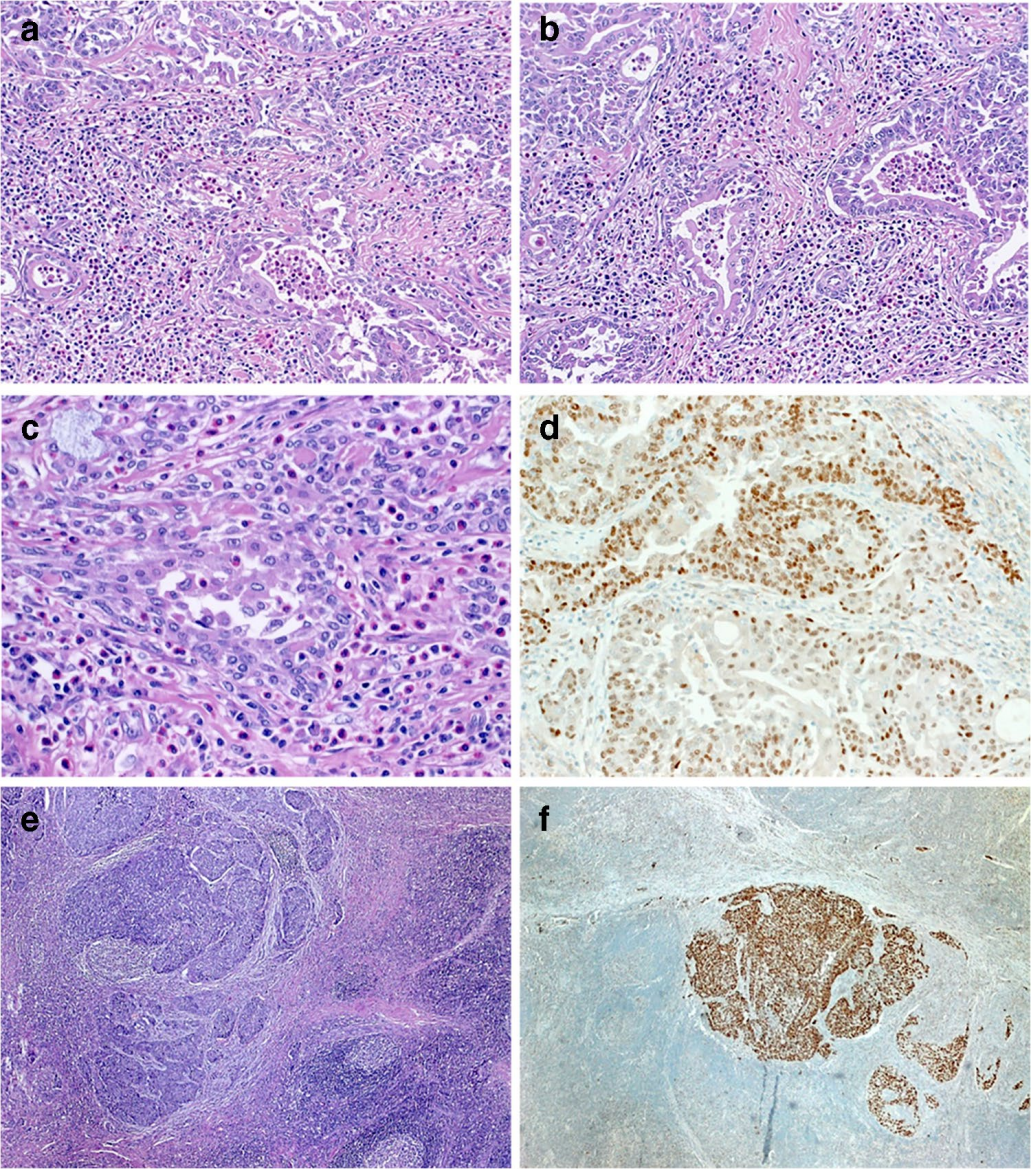
The SMECE showed infiltrating nests and irregular branching gland-like structures amid a prominent mixed eosinophil-rich inflammatory infiltrate with fine-reticular fibrosis (**a**, **b**). **c** Bland cytology is seen at high power. **d** p63 was variably expressed in majority of cells. **e** The lymph node metastases of the SMECE were less differentiated and predominantly basaloid but lacked squamous foci and were negative for the NUT IHC. **f** Diffuse expression of p63 in the nodal metastasis of SMECE

A 42-year-old woman presented in December 2018 with a slowly progressive, painful right neck swelling. Magnetic resonance imaging demonstrated a well-circumscribed mass occupying the right neck from the submandibular gland to the jugulum and infiltrating the right thyroid lobe with displacement of the trachea. Biopsy was obtained followed by right-sided hemithyroidectomy and lymph node dissection. Postoperative staging PET/CT scan did not show distant metastasis or other tumor manifestations. Histology was consistent with SMECE (Fig. 1).

**Fig. 2:**
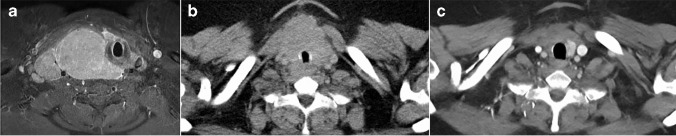
**a** MRI imaging showing a well-circumscribed pronounced mass occupying the right cervical region with infiltration of the thyroid bed and displacement of the trachea (corresponding to the NUT carcinoma). **b** CT imaging showing significant disease response after completion of definitive radiochemotherapy. **c** CT imaging on the last follow-up examination without signs of locoregional recurrence

**Fig. 3:**
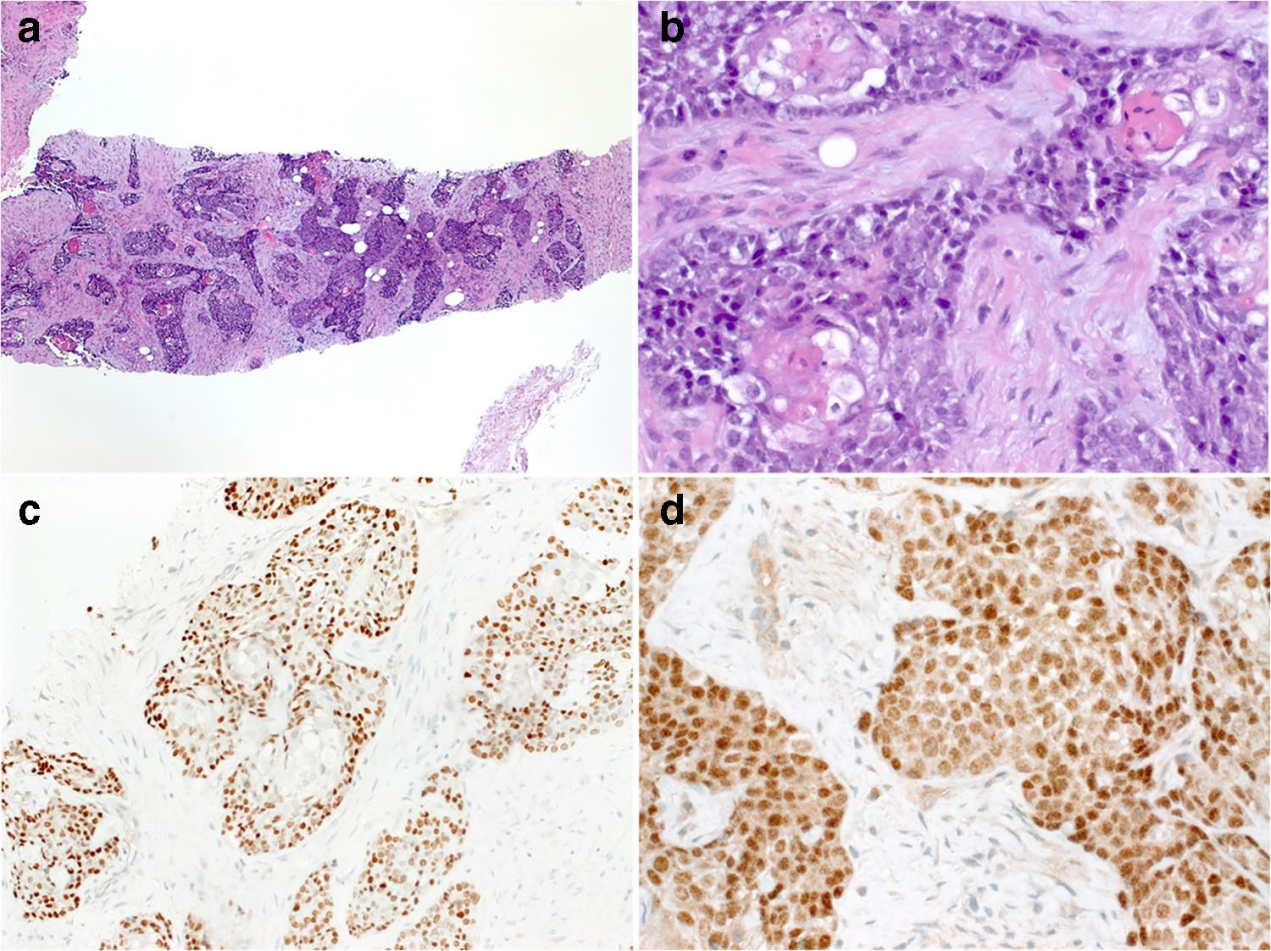
The core needle biopsy of the NUT carcinoma showed communicating nests and strands of monomorphic undifferentiated basaloid cells (**a**) with multiple foci of abrupt squamous differentiation (**b**), expression of p63 (**c**), and homogeneous nuclear reactivity with the NUT monoclonal antibody (**d**)

Six months later, in May 2019, the patient presented with an extensive locoregional recurrence encasing and compressing the trachea (Fig. 2a), interpreted as recurrent SMECE. However, core needle biopsies showed features of NUT carcinoma (Fig. 3). Definitive concurrent radiochemotherapy was recommended after case discussion at our interdisciplinary meeting. The tumor and the surrounding structures were irradiated with up to 70 Gy. Chemotherapy was administered with cisplatin 20 mg/m^2^ BSA (body surface area) days 1–5 and 29–33 and etoposide 90 mg/m^2^ BSA days 2–4 and 30–32. Partial disease remission was seen on post-interventional imaging (Fig. 2b). Treatment with molibresib (80 mg/day), a “small molecule” bromodomain and extra-terminal domain (BET) protein inhibitor, was initiated as a part of a “compassionate use” program. Four weeks later, treatment had to be terminated because of adverse effects (liver toxicity, thrombocytopenia, and nausea). At last follow-up (18 months since diagnosis of the NUT carcinoma and 12 months after completion of treatment), the patient was tumor-free on imaging (Fig. 2c).

## Materials and methods

Tissue specimens were fixed in formalin and processed routinely for histopathology. Immunohistochemistry (IHC) was performed on 3-µm sections using a fully automated system (“Benchmark XT System,” Ventana Medical Systems Inc., 1910 Innovation Park Drive, Tucson, AZ, USA) and the following antibodies: cytokeratin (AE1/AE3, 1:40, Zytomed), CK7 (OV-TL, 1:1000, Biogenex), p63 (SSI6, 1:100, DCS), S100 protein (polyclonal, 1:2500, Dako), synaptophysin (SY38, 1:50, Dako), thyroglobulin (2H11 + 6E1, RTU, Cell Marque), TTF1 (8G7G3/1, dilution, 1:500, Zytomed), SMARCB1 (MRQ-27, 1:50, Zytomed), IgG4 (HP6025, 1:200, BioRad), and anti-NUT antibody (clone C52B1, 1:45, Cell Signaling). Normal testis was used as positive control for NUT IHC.

### Next-generation sequencing and FISH testing

Molecular analyses for gene fusions (TruSight RNA Fusion Panel, Illumina, Inc., San Diego, CA, USA), gene mutations (TruSight 170 DNA Panel, Illumina), and NUT-FISH testing were performed as described previously [8].

## Results

### Pathological findings

The thyroidectomy specimen revealed an infiltrating epithelial neoplasm with variable epidermoid and adenoid growth pattern within an extensive fibroinflammatory stroma (Fig. 1a, b). The inflammatory infiltrate was predominantly eosinophilic and lymphoplasmacytic (Fig. 1c). The background thyroid showed variable chronic lymphocytic thyroiditis. IHC showed heterogeneous reactivity with CK7 and p63 (Fig. 1d), while TTF1, thyroglobulin, S100, and the NUT antibody were negative. Two regional nodes contained metastatic deposits showing solid basaloid morphology lacking squamous foci. They expressed diffusely p63 (Fig. 1e, f), but not the NUT antibody. The surrounding lymph node parenchyma showed prominent follicular hyperplasia and mixed eosinophil-rich inflammatory infiltrate. High numbers of IgG4-positive cells were seen in both the primary tumor and regional nodes (not shown).

The core needle biopsy of the recurrent mass revealed monomorphic undifferentiated basaloid cells arranged into communicating nests and strands with multiple foci of abrupt squamous differentiation (Fig. 3a, b). The IHC revealed homogeneous expression of p63 (Fig. 3c) and diffuse granular or punctate nuclear reactivity with the monoclonal anti-NUT antibody (Fig. 3d).

### Molecular findings

The primary SMECE revealed no pathogenic abnormalities; in particular, no *NUTM1* or other gene fusions were detected and no pathogenic mutations in *BRAF* or other genes were found. On the other hand, the needle biopsy of the recurrent mass showed the *NSD3-NUTM1* fusion. Exon 7 (breakpoint: chr8:38,184,248) of *NSD3* was fused to exon 3 (breakpoint: chr15:34,640,166) of *NUTM1*. Other pathogenic sequence variants were not detected. Due to the unexpected diagnosis of two rare entities in same patient, the DNA sequence was checked for pleomorphisms as surrogate for common origin from same subject. Shared variants were detected confirming origin of both samples from the same patient. The NUT-FISH revealed translocation signals in majority of tumor cell nuclei (not shown).

## Discussion

We herein describe a unique case of two exceptionally rare malignancies (one indolent and another highly aggressive) occurring sequentially at same anatomic site (median neck and thyroid) within a 6-month interval, hence closely mimicking recurrence of the same entity. We are not aware of any previous report on such an occurrence. SMECE is an exceedingly rare thyroid-specific low-grade malignancy with low but certain metastatic potential [2, 3]. It is distinct from salivary mucoepidermoid carcinoma histologically and by lack of *MAML2* fusions [2, 3]. Molecular studies failed to identify driver mutations in SMECE [3], except 2 recent cases with *BRAF V600E* mutations (both combined with a papillary carcinoma component [9]) and one with a *APC* variant of unknown significance (c.4073c > T; p.Ala1358Val), not described in thyroid neoplasms before [10].

NUT carcinoma has recently been reported to occur in diverse non-midline structures including the lateralized organs in the head and neck, thorax, and abdomen [11, 12]. The majority of cases (70%) harbor *BRD4-NUTM1* and rarely *BRD3-NUTM1* and *NSD3-NUTM1* fusions [4, 5, 11–13].

In the current case, a *NSD3-NUTM1*-rearranged carcinoma with classical features of NUT carcinoma was diagnosed in the thyroid bed following a recently resected SMECE. The clinical diagnosis was recurrent SMECE, but biopsy showed NUT carcinoma. Review of the previous specimen confirmed diagnosis of SMECE without features suggestive of a hybrid neoplasm. In such a scenario, it is mandatory to verify both diagnoses by molecular tools and to rule out specimen mislabelling. Diagnosis of independent SMECE and NUT carcinoma was verified by histological and immunohistochemical features and demonstration of the *NUTM1* fusion in the NUT carcinoma and its absence in the SMECE. Shared non-pathogenic variants in both specimens confirmed their origin from same patient.

Origin of a specific neoplastic entity from another pre-existing entity is well-known in germ cell neoplasia, where somatic-type malignancies of different types may be encountered post-germ cell neoplasms. However, origin of a translocation-driven malignancy from another is very unusual. We carefully reviewed the SMECE for any subtle NUT carcinoma-like component but could not identify any. The nodal metastases of the SMECE showed variable less differentiated solid basaloid morphology in addition to other features of SMECE but lacked features seen in the NUT carcinoma. Likewise, the NUT IHC was negative in the SMECE and its metastases. Although coincidental occurrence would not be excluded, several points strongly argue for a histogenetic relationship between the two entities. In particular, both entities are exceedingly rare making their coincidence very unlikely. Rare fusion-positive neoplasms such as salivary mucoepidermoid or secretory carcinoma have been reported in post-irradiation setting for other malignancies [14]. However, the time interval between both diagnoses in the current case is too short (6 months) and no irradiation was performed to act as potential explanation for the development of NUT carcinoma as a secondary malignancy.

It is possible, albeit merely speculative, that the *NUTM1* fusion has occurred as a second hit in a subclone of the SMECE that was missed on sampling (or not yet manifested morphologically) and then presented as overselected short-term extensive local recurrence. Finally, SMECE has been associated with papillary thyroid carcinoma (PTC), either concurrently or metachronously [2, 3, 9]. Concurrent PTC has been usually located away from the SMECE [2, 3]. A case of anaplastic thyroid carcinoma harboring a 30% SMECE component in addition to a PTC focus has been reported recently [15]. Another combined PTC-SMECE with *BRAF V600E* mutation recurred as anaplastic carcinoma in the thyroid bed 11 months later [9]. High-grade transformation (dedifferentiation) of SMECE into anaplastic carcinoma might represent an analogous phenomenon as in our current case, albeit of different molecular pathogenesis.

In summary, this case highlights sequential origin of two exceptionally rare entities at same anatomic site and underlines the necessity of sampling unexpectedly aggressive recurrences of otherwise indolent malignancies.
